# Screening and identification of nucleocapsid protein-nanobodies that inhibited Newcastle disease virus replication in DF-1 cells

**DOI:** 10.3389/fmicb.2022.956561

**Published:** 2022-07-27

**Authors:** Wenqi Fan, Pinpin Ji, Xuwen Sun, Min Kong, Ning Zhou, Qiang Zhang, Ying Wang, Qianqian Liu, Xiaoxuan Li, En-Min Zhou, Qin Zhao, Yani Sun

**Affiliations:** ^1^Department of Preventive Veterinary Medicine, College of Veterinary Medicine, Northwest Agriculture and Forestry University, Xianyang, China; ^2^Scientific Observing and Experimental Station of Veterinary Pharmacology and Diagnostic Technology, Ministry of Agriculture, Yangling, China

**Keywords:** nanobody, Newcastle disease virus, nucleocapsid protein, viral replication, inhibition

## Abstract

Newcastle disease (ND) is an acute and highly contagious infectious disease found in poultry. Although commercial ND virus (NDV) vaccines are universally used, some case reports persistently documented vaccination failure. Therefore, novel strategies are still required to control the occurrence of the disease in chickens. Recently, nanobodies (Nbs), which have the advantages of small molecular weight and low production costs, have been shown to be promising therapeutics against viral infection. In the present study, a total of 16 Nbs against NDV nucleocapsid protein (NP) were screened from two libraries against NDV using phage display technology. Of the 16 screened Nbs, eight were prevented from binding to NDV NP protein through administering positive chicken sera for anti-NDV antibodies, indicating that the epitopes recognized by these eight Nbs were able to induce the immune response after the chickens were infected with NDV stock. Subsequently, transfection assay, construction of recombinant DF-1 cells capable of expressing different nanobodies and viral inhibition assay were used to screen the nanobodies inhibiting NDV replication. The results demonstrated that Nb18, Nb30, and Nb88 significantly inhibited the replication of Class I and different genotypes of Class II NDV strains in DF-1 cells when they were expressed in the cytoplasm. Collectively, these nanobodies provided new tools for researching the functions of NDV NP protein and may be used as a novel strategy for designing drugs against NDV infection in chickens.

## Introduction

Newcastle disease (ND) is an acute and highly contagious infectious disease caused by Newcastle disease virus (NDV) that affects poultry. The disease is found worldwide, and is prevalent in many countries, mainly those in the African, Asian and American continents (Dmitriev et al., 2016). Since the 1950s, live and inactivated NDV vaccines have gradually become the main means for the prevention and control of ND. However, the increasing number of NDV variants that have been identified indicates that the genetic diversity of NDV is expanding (Bashir et al., 2018). The genetic differences consequently reduce the protective efficiency of the vaccines (Miller et al., 2007). Therefore, it is necessary to develop novel strategies for the prevention and treatment of NDV infection in poultry.

NDV is a single-stranded, non-segmented and negative-sense RNA virus. The complete genome encodes six proteins, including the nucleocapsid (NP), phosphoprotein (P), matrix (M), fusion (F), hemagglutinin-neuraminidase (HN) and large (L) proteins (Nagai et al., 1989). NP is the most abundant protein in virus particles, and serves to protect viral RNA from degradation by directly packaging the viral genome (Zhan et al., 2020). It also participates in forming ribonucleoproteins (RNPs) for viral replication with the proteins P and L, and genomic RNA (Dortmans et al., 2010). Therefore, NP is required for viral RNA transcription, replication and assembly. It has been established that the amino acid identity between Class I and Class II NDV NP is about 90% (Jahanshiri et al., 2005). Combined with the knowledge that NP protein functions in viral replication and that it is highly conserved, this suggests that NP may be an attractive target for drug development against different NDV strains.

Nanobodies (Nbs) are found in the variable region (VHH domains) of the *Camelidae* heavy chain antibodies (HcAbs) with a molecular weight of approximately 13–15 kDa, which are the smallest known antibody fragments with complete antigenic binding function (Rahbarizadeh et al., 2011). Owing to their small molecular weight, Nbs possess good tissue translocation and pharmacokinetic properties (Dmitriev et al., 2016). The characteristic of having only one domain also makes it possible to easily modify the Nbs, or to arrange them in tandem with other proteins, or Nbs to form new fusion proteins or polyvalent Nbs with low production costs (Els Conrath et al., 2001). Based on these advantages, the nanobodies have been used to design drugs against the disease in humans and animals. Previously, it has been reported that Nbs can be used as preventative and treatment drugs of anti-respiratory viruses, and they can be administered *via* aerosol (Nambulli et al., 2021).

In the present study, Nbs against NDV NP were screened from a constructed VHH library. Three Nbs expressed intracellularly in the cytoplasm were shown to inhibit the replication of different NDV strains in Chicken embryo fibroblast (DF-1) cells. Taken together, the results presented in this study have provided some nanobodies for researching the functions of NDV NP and valuable new information for the design of follow-up anti-NDV drugs.

## Materials and methods

### Ethics statement

All experiments procedures used in this study were performed based on the Guide for the Care and Use of Laboratory Animals of the Northwest Agriculture and Forestry University (NWAFU). The animal protocols were approved by the IACUC of NWAFU (approval no. 20200016/04).

### Cells, viruses and vectors

Human embryonic kidney epithelial (HEK-293 T) and DF-1 cell lines were both cultured in Gibco® DMEM (Thermo Fisher Scientific, 12,800,017), that the HEK-293 T supplemented with 10% (v/v) fetal bovine serum (Thermo Fisher Scientific, 10,091–148), and the DF-1 cells were supplemented with 5% (v/v) fetal bovine serum, at 37°C in an atmosphere of 5% CO_2_.

The NDV stock lentogenic LaSota strain (genotype II of Class II) was propagated in 9- to 11-day-old specific-pathogen-free (SPF) chicken embryos (Wang et al., 2015). In addition, the avirulent LaSota NDV carrying the gene of green fluorescence protein (NDV-GFP) was propagated in Vero cells (Kim and Samal, 2010; Lin et al., 2021). NDV virulent strain F48E9 (genotype IX of Class II; GenBank accession number, MG456905), clinical virulent strain JS/17 (genotype VII of Class II; GenBank accession number, MT811598.1), clinical virulent strain sx10 (genotype VI of Class II; GenBank accession number, KC853020) and Class I reference strain QH-1 (GenBank accession number, KT223818) kindly gifted by Prof. Sa Xiao and Prof. Zengqi Yang were also propagated in the SPF chicken embryos and used in the present study.

To produce a fusion protein between Nb and horseradish peroxidase (HRP), the pCMV-N1-HRP vector was constructed using pCMV-N1-EGFP (Takara Biological) as a template (Sheng et al., 2019). The lentiviral vector pLVX-IRES-ZsGreen1 and two helper packaging plasmids, psPAX2 and pMD2.0G, were used to assemble the recombinant lentivirus expressing Nb (Liu et al., 2015).

### Serum samples

To identify the epitopes recognized by the Nbs existing in the natural NDV particles, the sera from the SPF chickens infected with the NDV LaSota strain were used.12 At 28 days post-inoculation, a total of 10 serum samples were collected from 10 SPF chickens infected with the NDV LaSota strain *via* the nasal route. The hemagglutination (HA) titer of the NDV stock was 2^12^, and each SPF chicken was inoculated with 80 μl of the NDV LaSota strain. In addition, these serum samples were shown to be positive for anti-NDV antibodies using the hemagglutination inhibition (HI) test.

### Screening of specific Nbs against NDV NP protein

The phage rescue and biopanning procedures were performed as described previously (Vincke et al., 2012). Two phage display VHH libraries were constructed in two previous studies, comprising a library against NDV NP proteins (3 × 10^9^) and one against natural NDV particles (3 × 10^8^; Vincke et al., 2012; Sheng et al., 2019). In the present study, the two libraries were mixed for screening Nbs against NDV NP protein. The helper phage M13KO7 (NEB, N0315S) was used to rescue the mixed library. For biopanning, the purified NDV NP protein (LaSota strain) was used as the coating antigen (Sheng et al., 2019). After three rounds of biopanning, the enrichment of specific phage particles was evaluated by polyclonal phage enzyme-linked immunosorbent assay (ELISA; Sheng et al., 2019). Subsequently, a total of 96 colonies were randomly picked and induced with isopropyl-β-D-thiogalactoside (IPTG; 1 mM) for expression of the Nbs. After 14 h of Nb expression, the bacterial solution was collected and placed at −80°C prior to freeze-thawing. After resuspension and a subsequent centrifugation step, the supernatant obtained was the crude extract of the recombinant Nb. These crude extracts containing the Nbs were tested by indirect ELISA using purified NDV NP protein as the coated antigen. All positive clones containing variable domains of the *Camelidae* HcAbs (VHH domains) were sequenced and grouped according to their complementarity-determining region 3 (CDR3) sequences. All the above operations are carried out as described previously with modifications (Sheng et al., 2019).

### Expression of Nbs with HRP-fusion proteins

In order to express the Nb-HRP fusion proteins against the NDV NP protein, the vector pCMV-N1-HRP modified from the commercial vector pCMV-N1-EGFP was used as previously described (Sheng et al., 2019). The VHH genes encoding the screened Nbs obtained from the pMECS vectors were cloned into the pCMV-N1-HRP vector using both the *Pst*I and *Not*I restriction enzymes for digestion. The positive recombinant plasmids were confirmed by sequencing. Subsequently, the positive plasmids were transfected into HEK-293 T cells using transfection reagent polyetherimide (PEI; Polysciences, 24,765–1) at 37°C for 6 h to produce the Nb-HRP fusion proteins. Following transfection for 3 days, the supernatant containing Nb-HRP fusion protein was collected and centrifuged at 4°C 1,500× *g* for 10 min to remove the cell debris. Direct ELISA was performed according to the procedures described in a previous study to confirm that the Nb-fusion proteins still specifically bind to the NDV NP protein (Sheng et al., 2019). In addition, competitive ELISA performed based on the method was used to assess whether the interaction of Nb-HRP fusion proteins with NDV NP protein was blocked by the positive chicken sera for anti-NDV antibodies (Sheng et al., 2019).

### Preliminary screening of Nbs inhibiting NDV replication

The recombinant plasmids containing the different VHH genes as described above were also transfected into DF-1 cells by PEI. At 12 h post-transfection, the DF-1 cells were inoculated with the recombinant NDV-GFP strain [0.01 multiplicity of infection (MOI)]. After infection for 24 h, the cells were fixed and were directly observed under a confocal laser scanning microscope (Leica). Where the fluorescence intensity in the transfected cells was significantly less, this signified that the Nbs may inhibit NDV-GFP replication in the cells and, consequently, these cells were selected for further evaluation.

### Establishment of recombinant DF-1 cell lines stably expressing Nb

In order to further evaluate the role of Nbs against NDV replication in the cells, the recombinant DF-1 cells stably expressing Nbs were designed and constructed using a lentiviral platform (TaKaRa Biological). Based on the manual instructions, the VHH genes encoding Nbs in the pCMV-N1-HRP vector were digested with *EcoR* I and *Xba* I (10 U/μL), and inserted into the pLVX-IRES-ZSgreen1 vector. The sequences of the primers used for the construction of the recombinant pLVX-NDV-NP-Nbx plasmids are shown in Table 1. The recombinant plasmids were confirmed by sequencing. Subsequently, the positive recombinant plasmids and two helper plasmids (psPAX2 and pMD2.0G) were co-transfected into HEK-293 T cells using PEI. After the cells had been transfected for 72 h, the supernatant containing the recombinant lentivirus was collected at 1,500× *g* for 10 min and used to infect the DF-1 cells. Subsequently, the recombinant DF-1 cells stably expressing Nbs were screened using a limited dilution method, according to a method described previously (Ji et al., 2020). The cells in each well were observed using fluorescence microscopy (Leica AF6000). The cells showed green with the expression of enhanced green fluorescent protein (EGFP) in the well were cultured at 37°C and passaged ten times. The recombinant DF-1 cells stably expressing Nb were identified *via* Western blotting essentially the same as that described in the section below, using anti-HA monoclonal antibody as primary antibodies. The recombinant DF-1 cell line stably expressing the Nb-EGFP fusion protein was subsequently named the DF-1^Nbx-EGFP^ cell line. The blank vector, pLVX-IRES-ZSgreen1, was used as a negative control, and the recombinant DF-1 cell lines that only stably expressed EGFP were also constructed as control cells.

**Table 1:** Primers used in this study.

Primers	Primer sequences (5′–3′)	Purpose
pLVX-Nbx-F (*EcoR* I)	GGATCTATTTCCGGTGAATTCATGGAGTCTGGGGGAGGCT	Constructions of recombinant pLVX-NDV-NP-Nbx plasmids
pLVX-Nbx-R (*Xba* I)	GGGATCCGCGGCCGCTCTAGATTATGAGGAGACGGTGACCTGG	
NDV-NP-F	GTRATGAGRAACCATGTTGC	qRT-PCR
NDV-NP-R	CACTCCTRTTGTTGAACTG	
NDV-NP-probe	GCAGGGAAACAGRATGAAGCCACA	

### Cell counting kit-8 assay

According to the manufacturer’s protocols, we analyzed and compared the growth curves of the recombinant cells with that normal DF-1 cells by Cell Counting Kit-8 (Solarbio CA1210). Cells were seeded and cultured at a density of 1 × 10^4^/well in 100 μl of medium into 96-well microplates (Corning). After for 12 h, CCK-8 reagent was added to each well and then cultured for 2 h. All experiments were performed in triplicate. The absorbance was analyzed at 450 nm and the replication of cells was expressed by the absorbance.

### Virus infection assay

To confirm that the Nbs targeted against NDV NP protein were able to inhibit virus replication, the recombinant DF-1 cell lines were infected with different NDV isolates (LaSota, F48E9, JS/17, sx10 and QH-1). Briefly, different recombinant DF-1 cell lines were cultured on 24-well cell plates at a density of 1 × 10^5^ cells per well. After the cells had been cultured for 12 h, they were infected with 0.01 MOI of different NDV isolates. At 1 h after the cells were infected, they were washed with phosphate-buffered saline (PBS; 0.1 M, pH 7.2) three times, and cell culture was allowed to proceed with fresh medium. The cytopathic effects (CPEs) of different cell lines were observed under an inverted microscope (100 μm, Leica AF6000). In addition, the infected cells and supernatants were separately collected for testing the viral amounts by indirect immunofluorescence assay (IFA), Western blotting, reverse transcription-quantitative PCR (RT-qPCR) and median tissue culture infectious dose (TCID_50_) assays.

### Indirect immunofluorescence assay

After the cells had been infected for 12 h with NDV, they were washed three times with PBS (0.1 M; pH 7.2) and fixed with 70% iced ethanol. After the cells were subsequently blocked with 1% BSA at 4°C for 12 h, they were incubated with anti-NDV NP monoclonal antibody (mAb; Qianxun Biological, Ab-005) diluted with 0.5% PBS’T [PBS with 0.5% Tween-20 (v/v)] for 1 h at 25°C. The cells were washed three times with 0.5% PBS’T again, and then the cells were incubated with FITC-goat anti-mouse IgG antibody (Jackson ImmunoResearch Laboratories, 115–095-146) at 25°C for 1 h. Finally, the cell nuclei were stained with 4′, 6′-diamidino-2-phenylindole (DAPI) for 20 min, and cells were observed under a Leica AF6000 fluorescence microscope (Leica Microsystems GmbH).

### Western blotting analysis

The infected cells were collected and lysed with NP40 lysis buffer (Beyotime Institute of Biotechnology, P0013F) at 4°C, according to the manufacturer’s instructions. After the lysates had been removed by centrifugation (4°C, 12,000 × *g* for 15 min), the supernatant was subjected to SDS-PAGE in a 12% polyacrylamide gel and transferred to a nitrocellulose membrane. Then the membrane was first blocked with the blocking buffer (PBS’T with 5% skimmed milk) at room temperature for 2 h. After washing the membranes three times with PBS’T, the membranes were incubated with anti-NDV NP (1:500) and GAPDH (1:1,000) mAbs at 4°C for 14 h. After a further wash with PBS’T, the membranes were incubated with HRP-labelled goat anti-mouse IgG (1:2,000) at room temperature for 1 h. After extensive washing, the ECL solutions (Thermo Fisher Scientific) were incubated for 1 min with the blots prior to detection of the protein bands. The bands were observed, and the levels of protein expression were quantified *via* densitometric scanning using densitometry scans from Image Lab (Version 5.1; Bio-Rad Laboratories).

### Reverse transcription-quantitative PCR

Total RNA was extracted from the infected cells, and 500 ng of RNA was reverse-transcribed into complementary deoxyribonucleic acid (cDNA) using an M-MLV reverse transcription reagent (Accurate Biology, AG11728). A StepOnePlus^™^ Real-Time PCR System (Applied Biosystems) was used for RT-qPCR analysis. The recombinant plasmid pMD-19 T-NDV-NP was used as a positive control to create the standard curve. After the plasmids had been converted into copies, nine concentration gradients (10^1^–10^9^) were tested, enabling the standard curve to be drawn.

The total volume of reaction solution was 15 μl, which comprised 7.5 μl of Premix Pro Taq™ HS mixture (Accurate Biology, AG11704), 2 μl of cDNA template, 0.3 μl of forward primers, 0.3 μl of reverse primers, 0.6 μl of primer probe, 0.3 μl of ROX Reference dye and 4 μl of water. The primer sequences used for RT-qPCR are shown in Table 1.

### TCID_50_ detection

The TCID_50_ of the supernatants from the infected cells were also examined. Briefly, the supernatant containing NDV (0.1 ml) was added to 0.9 ml of medium for a 10-fold series dilution. After the DF-1 cells were plated on to 96-well cell plates, dilutions of 10^−1^ to 10^−8^ were used to infect the cells (8 wells for each dilution). Meanwhile, eight wells were used as a negative control. The CPEs were observed, and the TCID_50_ values were calculated using the Reed-Munch method (Pizzi, 1950).

### Statistical analysis

All experiments were performed at least three times. Statistical significance was determined by one-way analysis of variance (ANOVA). A *p*-value < 0.05 was considered to indicate a statistically significant value.

## Results

### Screening and identification of anti-NDV NP specific Nbs

**Figure 1:**
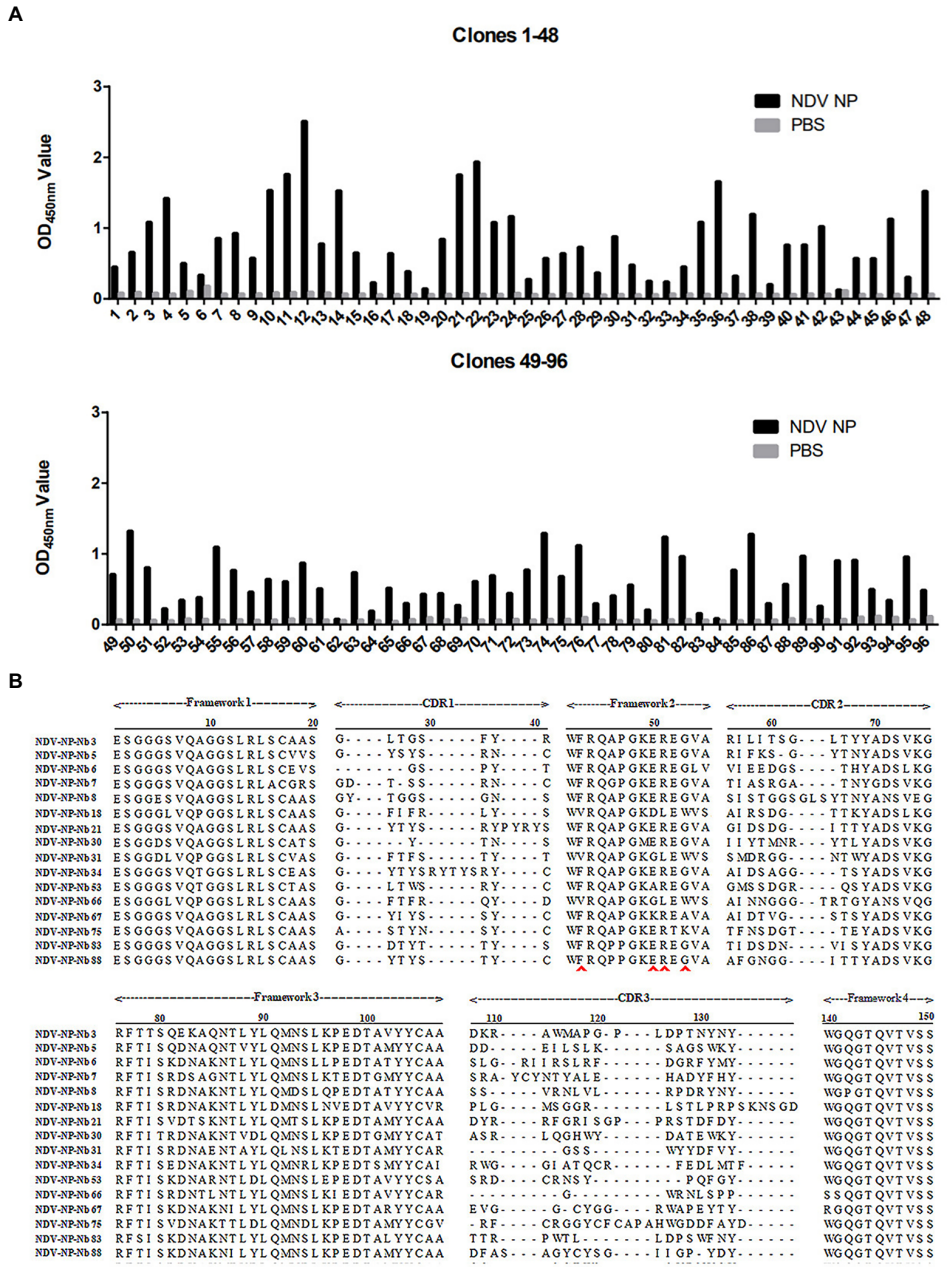
Screening of specific nanobodies against the Newcastle disease virus (NDV) NP protein. **(A)** Detection of the periplasmic extracts containing recombinant nanobodies from 96 clones reacting with NDV NP proteins with indirect ELISA. **(B)** Alignment of amino acid sequences of the 16 screened nanobodies. The frameworks and CDRs were labelled according to that described in a previous study, and the residues at positions 37, 44, 45, and 47 that are characteristic of nanobodies are indicated by the red arrows.

NP recombinant protein was used as coated antigen to screen specific nanobody. During the three rounds of screening, the phage recovery rate increased gradually (Table 2). After the third round of screening, the P/N value could reach 2.23 × 10^6^ (Table 2), indicating that the specific recombinant phages were enriched significantly. The periplasmic extracts containing Nbs were extracted from the 96 colonies and tested by indirect ELISA. The results showed that 86 of the colonies reacted specifically with the NDV NP proteins, which optical density (OD) value on 450 nm more than 3 times of PBS control is considered positive (Figure 1A). After they were sequenced, 16 unique Nbs against NDV NP protein were obtained based on the CDR3 regions of VHH genes, and these were separately named as NDV-NP-Nb3, -Nb5, -Nb6, -Nb7, -Nb8, -Nb18, -Nb21, -Nb30, -Nb31, -Nb34, -Nb53, -Nb66, -Nb67, -Nb75, -Nb83 and -Nb88 (Figure 1B).

**Table 2:** Enrichment of phage particles carrying NDV-NP-specific Nbs.

Round of banning	Input phage (pfu/well)	P output phage (pfu/well)	N output phage (pfu/well)	Recovery (P/input)	P/N
1st round	5 × 10^10^	1.15 × 10^6^	3.11 × 10^6^	2.3 × 10^−3^	0.369
2nd round	5 × 10^10^	2.09 × 10^6^	4 × 10^3^	4.18 × 10^−3^	522.5
3rd round	5 × 10^10^	2.23 × 10^6^	0	4.46 × 10^−3^	2.23 × 10^6^

### Nb-HRP fusion proteins specifically binding to NDV NP protein

**Figure 2:**
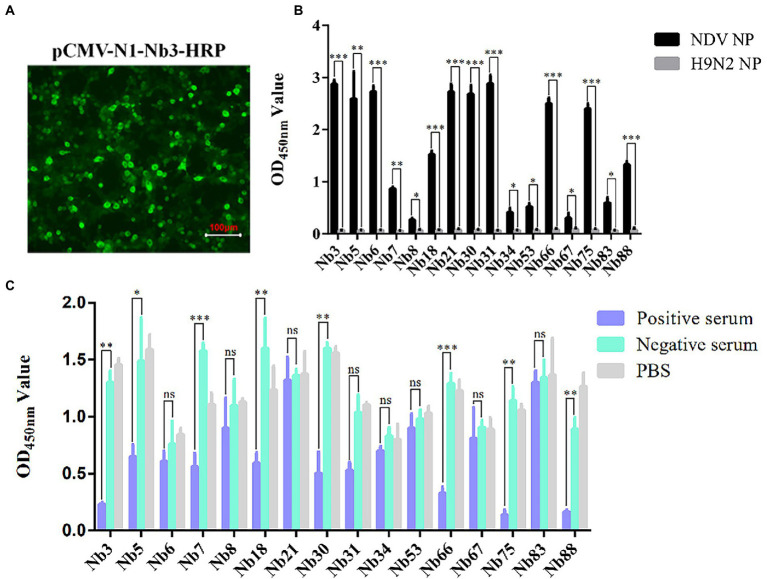
Secreted expression of nanobodies with HRP fusion proteins against NDV NP protein in the HEK-293 T cells. **(A)** Detection of nanobodies with HRP fusion protein expressed in the HEK-293 T cells *via* IFA using anti-HA mAbs as primary antibodies. HEK-293 T cells transfected with pCMV-N1-Nb13-HRP plasmids were selected and the cells transfected with other recombinant nanobody plasmids were treated in a similar manner. **(B)** Supernatants containing 16 nanobodies with HRP fusion proteins against NDV NP proteins were detected using direct ELISA. **(C)** Interactions between nanobodies with HRP fusion proteins and NDV NP proteins were blocked by the positive chicken sera for antibodies against NDV with competitive ELISA. The results were performed in triplicate and data are presented as the mean ± SD. ^*^p < 0.05; ^**^p < 0.01; ^***^p < 0.005 (two-tailed Student’s *t*-test).

After the recombinant plasmids were sequenced, the 16 VHH genes encoding Nbs were successfully inserted into the pCMV-N1-HRP vector (data not shown). After the positive plasmids were transfected into the HEK-293 T cells, IFA was performed to confirm that these Nbs had been successfully expressed in the cells (Figure 2A). Moreover, the direct ELISA results showed that the supernatant containing Nb-HRP fusion proteins still specifically reacted with NDV NP protein (Figure 2B), indicating that the fusion proteins had been secreted into the supernatant (OD value more than 3 times of H9N2 NP control is considered positive). These Nb-HRP fusion proteins in the supernatants were separately named NDV-NP-Nb3-HRP, -Nb5-HRP, -Nb6-HRP, -Nb7-HRP, -Nb8-HRP, -Nb18-HRP, -Nb21-HRP, -Nb30-HRP, -Nb31-HRP, -Nb34-HRP, -Nb53-HRP, -Nb66-HRP, -Nb67-HRP, -Nb75-HRP, -Nb83-HRP and -Nb88-HRP. The results of a competitive ELISA showed that eight Nbs were significantly blocked by the positive chicken sera for anti-NDV antibodies, and were thereby unable to react with NDV NP protein (Figure 2C). These results suggested that the epitopes recognized by these Nbs induced antibody responses after NDV had infected the chickens. Subsequently, these eight Nbs were selected for virus inhibition assay.

### Initial identification of Nbs against NDV NP inhibiting virus replication

**Figure 3:**
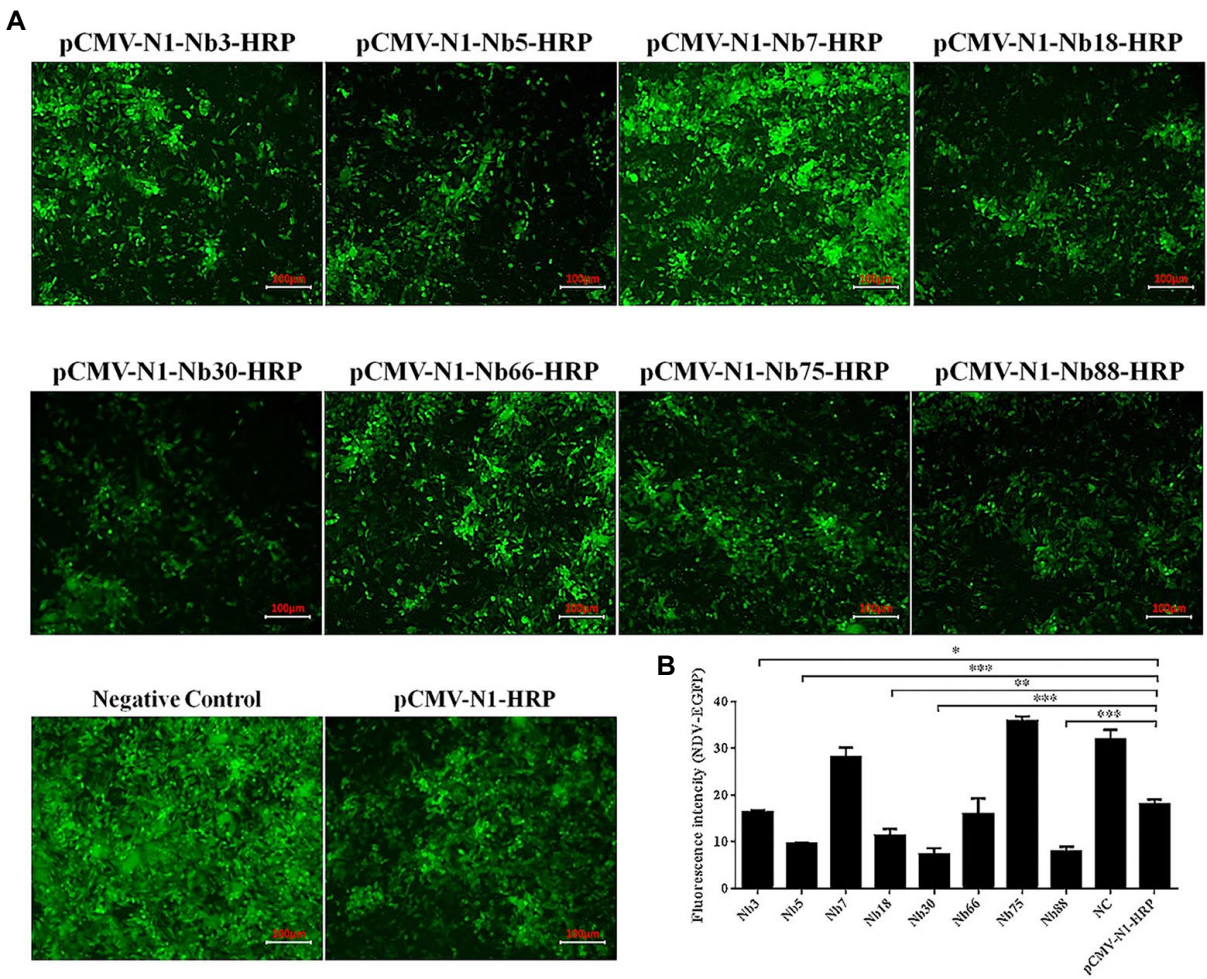
Preliminary analysis of intracellular expressing nanobodies against NDV NP proteins inhibiting replication of the recombinant NDV-GFP strain in DF1 cells. **(A)** Results of the intracellular GFP experiments in DF-1 cells infected with recombinant NDV-GFP strain. **(B)** Fluorescence intensity of the NDV-GFP was shown. The results were performed in triplicate and data are presented as the mean ± SD. ^*^p < 0.05; ^**^p < 0.01; ^***^p < 0.005 (two-tailed Student’s *t*-test).

The eight Nbs were used initially to investigate the inhibition of NDV replication in DF-1 cells. After the eight recombinant plasmids pCMV-N1-Nb3-HRP, -Nb5-HRP, -Nb7-HRP, -Nb18-HRP, -Nb30-HRP, -Nb66-HRP, -Nb75-HRP, and -Nb88-HRP had been transfected into DF-1 cells for 12 h, these cells were subsequently infected with the NDV-GFP strain. Cells transfected with the pCMV-N1-HRP vector served as a negative control and cells that were infected with only NDV-GFP were used as a blank control. Results showed that Nb3, Nb5, Nb18, Nb30, and Nb88 were able to significantly inhibit replication of the NDV-GFP strain in the DF-1 cells (Figure 3A). In addition, average fluorescence intensity (Fluorescence intensity = Integrated Density/Area) also showed that the five Nbs inhibit NDV-GFP replication in DF-1 cells (Figure 3B).

### Recombinant DF-1 cell lines stably expressing Nb3, Nb5, Nb18, Nb30 and Nb88

**Figure 4:**
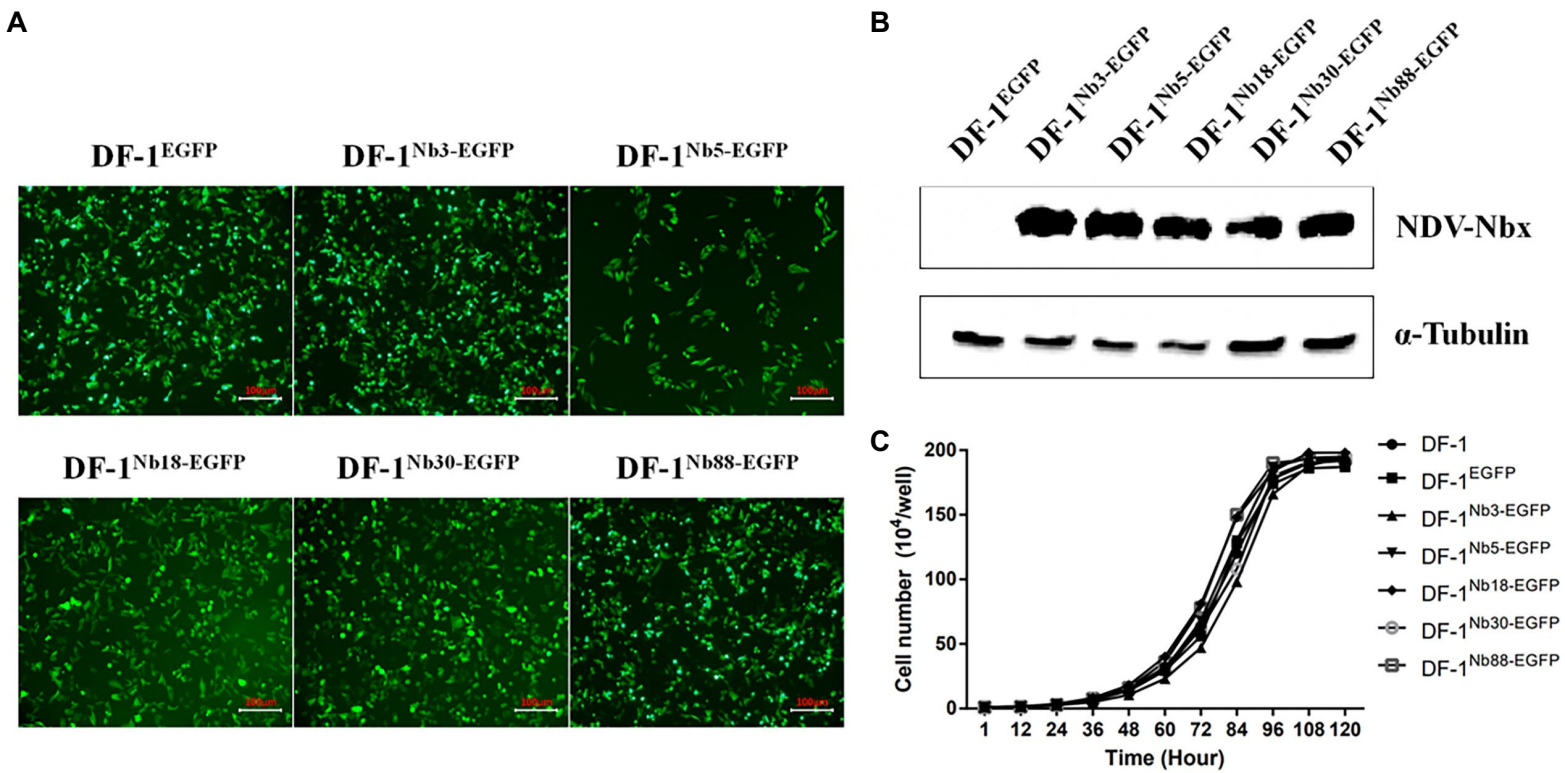
Establishment of recombinant DF1 cell lines stably intracellularly expressing enhanced green fluorescent protein (EGFP), Nb3-EGFP, Nb5-EGFP, Nb18-EGFP, Nb30-EGFP, and Nb88-EGFP. **(A)** All six recombinant DF1 cells lines stably expressing EGFP proteins were observed under a fluorescence microscope. **(B)** Detection of nanobodies with HA and EGFP tags in the recombinant DF1 cell lines *via* Western blotting analysis using anti-HA mAbs as primary antibodies. **(C)** Analysis of cell growth curves of the six recombinant DF1 cell lines. These were determined for cells seeded in 96-well plates (5 × 10^4^ cells/well). Assays were performed in triplicate, and data are expressed as the mean ± SD.

To further confirm that the Nb3, Nb5, Nb18, Nb30, and Nb88 were able to inhibit NDV replication in the DF-1 cells, recombinant DF-1 cells stably expressing these Nbs were constructed *via* lentivirus transduction assay (Sheng et al., 2019). The six recombinant DF-1 cell lines (including the negative control cell line) with green fluorescence were screened using the finite dilution method, and the cells were imaged using fluorescence microscopy. The six recombinant DF1 cells were named DF-1^EGFP^ (negative control cells), DF-1^Nb3-EGFP^, DF-1^Nb5-EGFP^, DF-1^Nb18-EGFP^, DF-1^Nb30-EGFP^ and DF-1^Nb88-EGFP^ (Figure 4A). Western blotting analysis showed that Nb3, Nb5, Nb18, Nb30, and Nb88 were successfully expressed in these recombinant cell lines (Figure 4B). In addition, the results of CCK-8 assay showed that no significant differences existed comparing the growth curves that were obtained for the six recombinant and normal DF-1 cells (Figure 4C).

### Recombinant DF-1 cell lines stably expressing Nbs inhibit NDV replication

**Figure 5:**
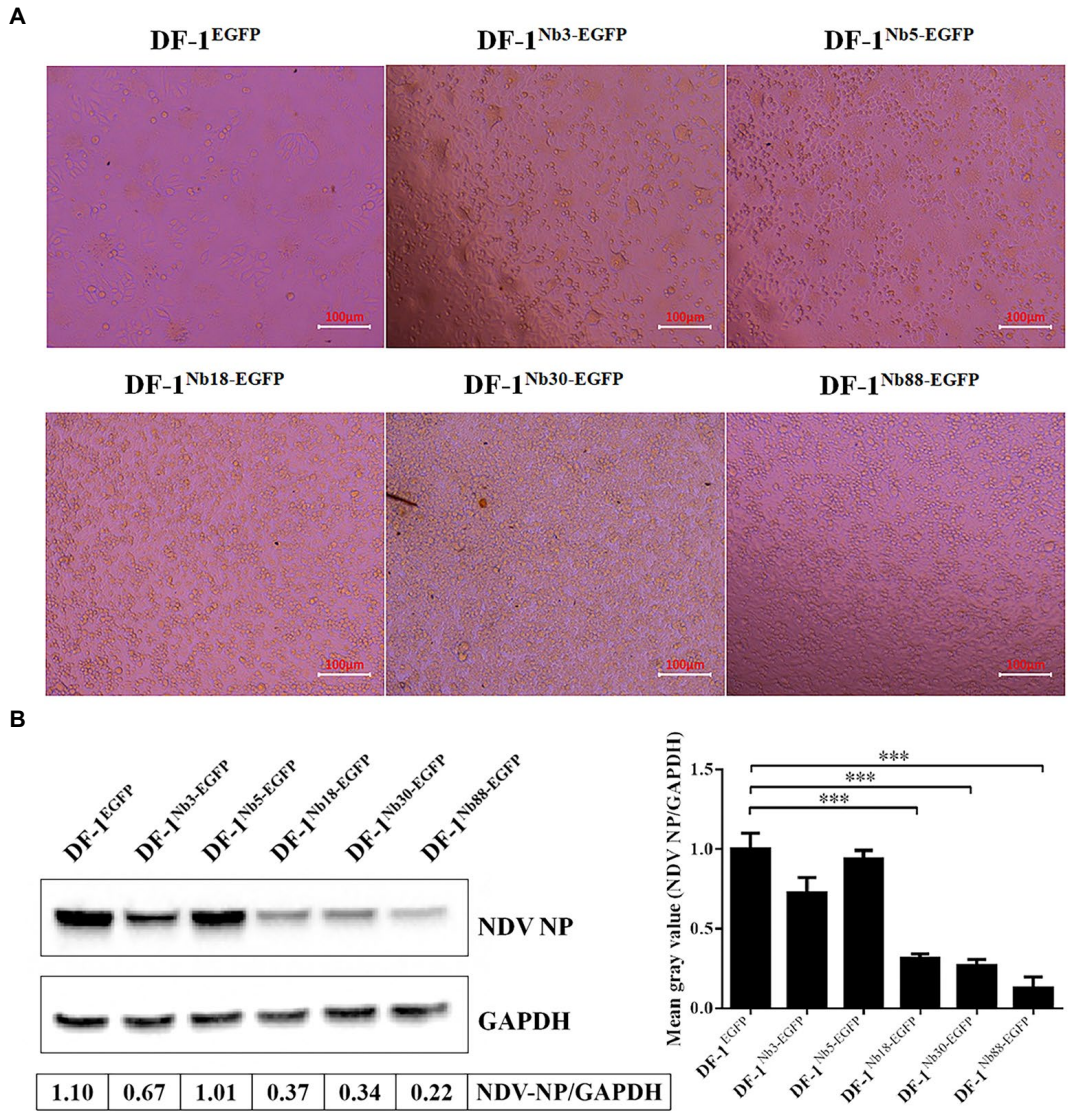
Analysis of the six recombinant DF1 cell lines inhibiting NDV LaSota strain replication. **(A)** Cytopathic effects of the six recombinant DF1 cell lines inoculated with 0.01 MOI of NDV LaSota strain. **(B)** Expression levels of NDV NP protein in different recombinant DF1 cell lines inoculated with NDV LaSota strain, as measured by Western blotting using anti-NDV NP mAbs as primary antibodies. The results were performed in triplicate and data are presented as the mean ± SD. ^*^p < 0.05; ^**^p < 0.01; ^***^p < 0.005 (two-tailed Student’s *t*-test).

After the six recombinant and normal DF-1 cells were inoculated with 0.01 MOI of NDV LaSota strain for 24 h, the CPEs were observed in the infected cells under an inverted microscope. The infected DF-1^EGFP^, DF-1^Nb3-EGFP^ and DF-1^Nb5-EGFP^ cells clearly showed CPEs, including syncytium formation and rupturing of the cells (Figure 5A). However, the infected DF-1^Nb18-EGFP^, DF-1^Nb30-EGFP^ and DF-1^Nb88-EGFP^ cells showed only slight CPEs (Figure 5A), indicating that the intracellularly expressed Nb18, Nb30, and Nb88 inhibited NDV replication. In addition, the results of the Western blotting analysis revealed that all five Nbs inhibited virus replication in the cells, and the inhibitory effects of the Nb18, Nb30, and Nb88 were more pronounced (Figure 5B). Therefore, the three recombinant cell lines DF-1-Nb18^EGFP^ -Nb30^EGFP^ and -Nb88^EGFP^ were selected to evaluate their ability to inhibit the replication of different NDV isolates.

### Recombinant DF-1-Nb18^EGFP^, -Nb30^EGFP^ and –Nb88^EGFP^ cell lines inhibit replication of different NDV strains

**Figure 6:**
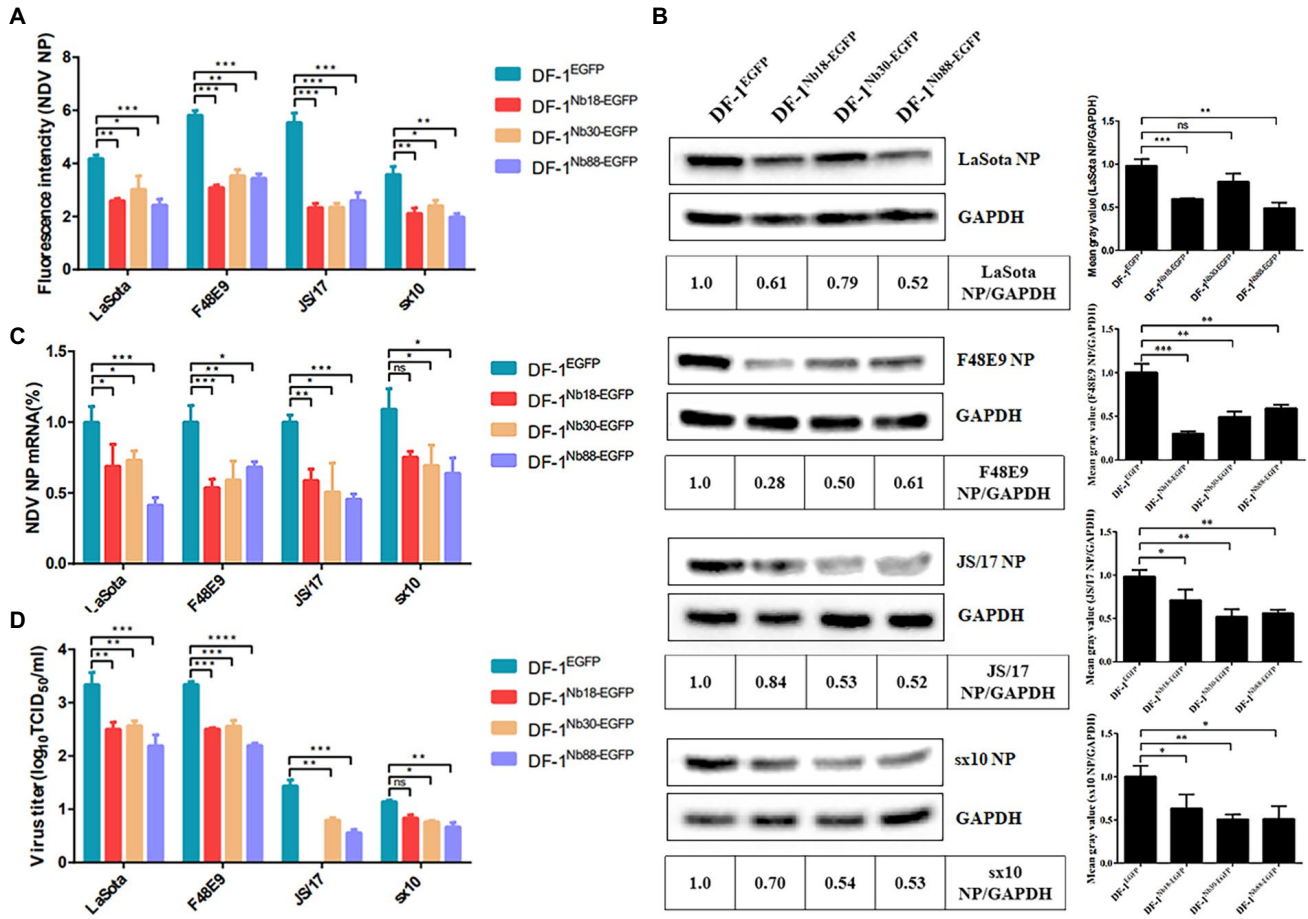
Analysis of the four DF1^EGFP^, DF1^Nb18-EGFP^, DF1^Nb30-EGFP^ and DF1^Nb88-EGFP^ cell lines inhibiting the replication of different Class II NDV strains, including LaSota, F48E9, JS/17 and sx10. **(A)** Mean gray values of indirect immunofluorescence detection of the different NDV strains after infection of the different cell lines are shown. **(B)** Detection of NDV NP proteins in the four recombinant DF1 cell lines inoculated with LaSota, F48E9, JS/17 and sx10 strains. **(C)** qRT-PCR to detect the transcription level of the different NDV strains NP protein mRNA in different cell lines. **(D)** Virus titers (TCID_50_) derived from the supernatant of the four recombinant DF1 cell lines inoculated with the different NDV strains. The results were performed in triplicate and data are presented as the mean ± the SD. *p < 0.05; **p < 0.01; ^***^p < 0.005 (two-tailed Student’s *t*-test).

The recombinant DF-1^Nb18-EGFP^, DF-1^Nb30-EGFP^ and DF-1^Nb88-EGFP^ cell lines that inhibited the replication of the different NDV strains were further analyzed. After the three cell lines were inoculated with different NDV isolates, IFA results showed that the intensity of red fluorescence in DF-1^Nb18-EGFP^, DF-1^Nb30-EGFP^ and DF-1^Nb88-EGFP^ cells was significantly less compared with that of the DF-1^EGFP^ cells for the four isolates from the different genotypes of Class II (Figure 6A). Fluorescence results are shown in Supplementary Figures S1, S2. Western blotting analysis also revealed that the three Nbs, Nb18, Nb30, and Nb88, all significantly inhibited NDV replication in DF-1 cells, and the inhibition rates for the F48E9 and sx10 strains reached approximately 50% (Figure 6B). The RT-qPCR results also showed that the relative levels of the mRNAs of NDV NP in the DF-1^Nb18-EGFP^, DF-1^Nb30-EGFP^ and DF-1^Nb88-EGFP^ cell lines were less than that found in the DF-1^EGFP^ cells (Figure 6C). In addition, the titers of supernatants from the DF-1^Nb18-EGFP^, DF-1^Nb30-EGFP^ and DF-1^Nb88-EGFP^ cells were less than that from the DF-1^EGFP^ cells, as determined from measuring the TCID_50_ values (Figure 6D). Taken together, these results showed that the intracellular expression of Nb18, Nb30, and Nb88 in DF-1 cells led to a significant inhibition in the replication of different NDV (Class II) strains.

**Figure 7:**
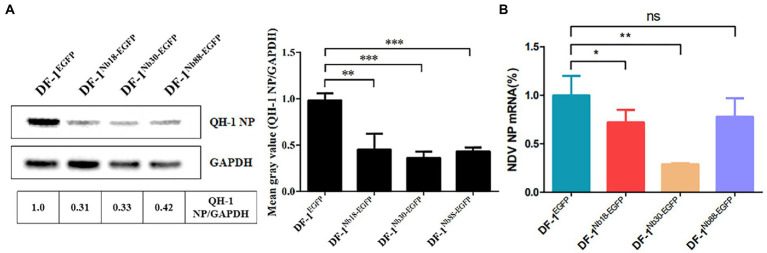
Analysis of the four recombinant DF1 cell lines inhibiting replications of Class I NDV strains (QH-1). **(A)** Detection of NDV NP proteins in the four recombinant DF1 cell lines inoculated with NDV QH-1 strain by Western blotting. **(B)** Detection of NDV NP mRNAs in the four recombinant cells inoculated with the QH-1 strain *via* RT-qPCR assay. The results were performed in triplicate and data are presented as the mean ± SD. *p < 0.05; **p < 0.01; ^***^p < 0.005 (two-tailed Student *t*-test).

To further analyze whether the three Nbs could inhibit replication of the NDV strain form Class I in DF-1 cells, the reference QH-1 strain was selected. The results of Western blotting and RT-qPCR assays showed that the protein and mRNA levels of NDV NP in DF-1^Nb18-EGFP^, DF-1^Nb30-EGFP^ and DF-1^Nb88-EGFP^ cells were lower compared with that in DF-1^EGFP^ cells, and the inhibitory effect mediated by Nb30 was relatively the highest (Figure 7).

## Discussion

Since mAbs have been used as therapeutic drugs (Oldham and Dillman, 2008) many mAbs have been approved by the U.S. Food and Drug Administration (FDA) and are available on the market (Oliveira et al., 2013). However, due to their complicated production processes and high costs, traditional antibodies have seldom been used in animals, especially livestock. Recently, Nbs, which possess the advantages of an easier production process and low production costs, have shown good prospects in terms of their applicability as drugs for animal husbandry, particularly for poultry (Steeland et al., 2016; Salvador et al., 2019). In the present study, a total of 16 Nbs targeted against NDV NP protein were screened, and eight of them were blocked from binding to NDV NP protein by positive chicken serum for anti-NDV antibodies. Subsequently, three of the Nbs (Nb18, Nb30, and Nb88) expressed in the cytoplasm were shown to be able to inhibit the replication of different NDV strains in DF-1 cells. To the best of the authors’ knowledge, this is the first time that intracellular Nbs have been reported to inhibit NDV replication in DF-1 cells. Furthermore, the study has provided a foundational basis for developing a novel strategy for combating NDV infection in chickens.

In spite of the fact that there is only one serum type, the NDV strains have evolved into twenty-one genotypes (Dimitrov et al., 2019). Currently, the NDV LaSota strain, belonging to genotype II, is still commonly used as the vaccine strain. However, certain studies have reported that the commercially available LaSota vaccine strain is not able to provide complete protection against infections caused by the other genotype NDV strains (Cornax et al., 2012). For example, since the late 1990s, genotype VII strains have been the dominant ones circulating in China (Liu et al., 2003; Qin et al., 2008). However, many immune failure cases have been reported following immunization of the chickens with the commercial LaSota vaccine strain in China (Xiao et al., 2012). Therefore, in order to control the disease in poultry more effectively, the development of a broad-spectrum vaccine or anti-viral agent is still being sought after. Compared with other viral proteins of NDV, the NP protein is highly conserved, and therefore provides an ideal target for drug design. In the present study, the viral inhibition assay showed that the intracellularly expressed Nb18, Nb30 and Nb88 were able to inhibit the replication of LaSota (genotype II of Class II), F48E9 (genotype IX of Class II), JS/17 (genotype VII of Class II), sx10 (genotype VI of Class II) and QH-1 (Class I reference strain) strains in DF-1 cells. These results suggested that the three Nbs may inhibit replication of the different genotypes of NDV *in vitro*. In the future, other genotypes of the NDV isolates will be studied to further corroborate these findings.

Previously published studies have demonstrated that the NDV NP protein fulfills important roles in viral replication (Xu et al., 2019). The protein is able to tightly bind to the viral genome RNA, and participates in the assembly of the virus RNP and viral particles. Therefore, if therapeutically administered agents were to be able to block these functions of NDV NP protein, they would be able to inhibit viral replication. Based on the findings of the present study, we surmise that the ability of Nb18, Nb30, and Nb88 to inhibit NDV replication may be explained by blockade of the above functions of NDV NP protein by these three Nbs. However, to fully identify the mechanism through which the three Nbs inhibit NDV replication, the epitopes recognized by the three Nbs, and the functions of motifs in these epitopes, will need to be further analyzed. Interestingly, viral inhibition assay showed that the intracellularly expressed Nb7 and Nb75 were able to significantly promote the replication of NDV in DF-1 cells. Similarly, it will be interesting to understand the mechanism through which these two Nbs promote NDV replication. In addition, the Nbs that were screened against NDV NP protein may serve as tools in order to investigate the functions of the protein.

The results obtained in the present study have shown that the efficiency with which the three Nbs inhibit replication of the different NDV strains is inconsistent. The underlying reason may be the difference in affinity of Nb for the NP proteins of the different NDV strains. In addition, our results also showed that the three Nbs could not completely inhibit NDV replication in the DF-1 cell lines. Our hypothesis is that the binding of Nbs with NDV NP protein is a dynamic process, and the complexes formed between NP protein and Nbs are not as strong as those of the complexes formed between traditional antibodies and antigens. Therefore, in the future, we aim to explore the expression of multiple Nbs fused together, in order to generate highly effective Nbs against NDV replication.

## Data availability statement

The original contributions presented in the study are included in the article/Supplementary Material, further inquiries can be directed to the corresponding authors.

## Ethics statement

The animal study was reviewed and approved by the Care and Use of Laboratory Animals of the Northwest Agriculture and Forestry University (NWAFU). The animal protocols were approved by the IACUC of NWAFU (approval no. 20200016/04).

## Author contributions

YS conceived the study and revised the paper. WF and PJ performed the experiments and wrote the paper. XS, MK, NZ, and QZn contributed to reagents and analysis tools. YW, QL, and XL contributed to the data analyses. All authors contributed to the article and approved the submitted version.

## Funding

This work was supported by grants from Science and Technology Program of Xi’an (20193033YF021NS021) to YS and National Key R&D Program of China (2016YFD0500800) and Tang Scholar of Cyrus Tang Foundation to QZo.

## Conflict of interest

The authors declare that the research was conducted in the absence of any commercial or financial relationships that could be construed as a potential conflict of interest.

## Publisher’s note

All claims expressed in this article are solely those of the authors and do not necessarily represent those of their affiliated organizations, or those of the publisher, the editors and the reviewers. Any product that may be evaluated in this article, or claim that may be made by its manufacturer, is not guaranteed or endorsed by the publisher.

## Supplementary material

SUPPLEMENTARY FIGURE S1 Schematic diagram of lentivirus vector PLVX-IRES-ZSgreen1.

SUPPLEMENTARY FIGURE S2 Detection of the different Class II NDV strains after infection of the different cell lines by IFA. NDV LaSota strain infected different cell lines (Upper left). NDV F48E9 strain infected different cell lines (Upper right). NDV JS/17 strain infected different cell lines (Lower left). NDV sx10 strain infected with different cell lines (Lower right).
