# Corrigendum: Screening and identification of nucleocapsid protein-nanobodies that inhibited Newcastle disease virus replication in DF-1 cells

**DOI:** 10.3389/fmicb.2022.1045834

**Published:** 2022-10-31

**Authors:** Wenqi Fan, Pinpin Ji, Xuwen Sun, Min Kong, Ning Zhou, Qiang Zhang, Ying Wang, Qianqian Liu, Xiaoxuan Li, En-Min Zhou, Qin Zhao, Yani Sun

**Affiliations:** ^1^Department of Preventive Veterinary Medicine, College of Veterinary Medicine, Northwest Agriculture and Forestry University, Xianyang, China; ^2^Scientific Observing and Experimental Station of Veterinary Pharmacology and Diagnostic Technology, Ministry of Agriculture, Yangling, China

**Keywords:** nanobody, Newcastle disease virus, nucleocapsid protein, viral replication, inhibition

In the original article, there was an error in **Materials and methods**, “*Cells, viruses and vectors”* Paragraph 2, where the source information for the NDV clinical strains was not stated.

This sentence previously stated:

“NDV virulent strain F48E9 (genotype IX of Class II; GenBank accession number, MG456905), clinical virulent strain JS/17 (genotype VII of Class II; GenBank accession number, MT811598.1), clinical virulent strain sx10 (genotype VI of Class II; GenBank accession number, KC853020) and Class I reference strain QH-1 (GenBank accession number, KT223818) were also propagated in the SPF chicken embryos and used in the present study.”

The corrected sentence appears below:

“NDV virulent strain F48E9 (genotype IX of Class II; GenBank accession number, MG456905), clinical virulent strain JS/17 (genotype VII of Class II; GenBank accession number, MT811598.1), clinical virulent strain sx10 (genotype VI of Class II; GenBank accession number, KC853020) and Class I reference strain QH-1 (GenBank accession number, KT223818) kindly gifted by Prof. Sa Xiao and Prof. Zengqi Yang were also propagated in the SPF chicken embryos and used in the present study.”

The authors apologize for this error and state that this does not change the scientific conclusions of the article in any way. The original article has been updated.

## Publisher's note

All claims expressed in this article are solely those of the authors and do not necessarily represent those of their affiliated organizations, or those of the publisher, the editors and the reviewers. Any product that may be evaluated in this article, or claim that may be made by its manufacturer, is not guaranteed or endorsed by the publisher.

