# Supplementary figures and images for: Screening and identification of nucleocapsid protein-nanobodies that inhibited Newcastle disease virus replication in DF-1 cells
Source: Front Microbiol. 2022 Jul 27;13:956561. doi: 10.3389/fmicb.2022.956561 (PMC9426676; doi:10.3389/fmicb.2022.956561)

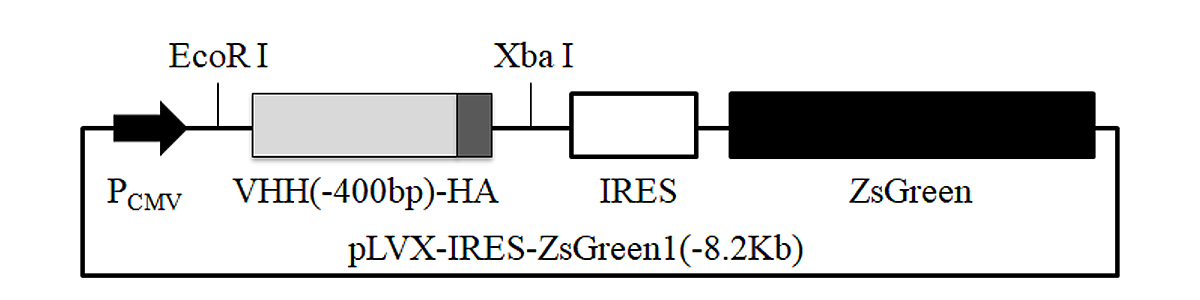

Supplement: SUPPLEMENTARY FIGURE S1 — Schematic diagram of lentivirus vector PLVX-IRES-ZSgreen1. [file Image_1.TIF]
